# Confronting Physical, Chemical and Temporal Biases in Experimental Algal Biology

**DOI:** 10.3390/ijms27198739

**Published:** 2026-09-30

**Authors:** Kunshan Gao, John Beardall

**Affiliations:** 1 State Key Laboratory of Marine Environmental Science, College of Ocean and Earth Sciences, Xiamen University, Xiamen 361102, China; 2 School of Biological Sciences, Monash University, Clayton, VIC 3800, Australia

**Keywords:** algae, cyanobacteria, methodological examination, phytoplankton, selective bias

## Abstract

Selective bias in algal research often leads to proposals of what are often unreliable causal relationships. Systematic errors mainly arise from ignoring chemical and physical environmental changes along with increased algal biomass during cultures. This paper examines three interacting bias dimensions using hypothetical cultures and literature examples. Selective bias in choosing basic parameters to measure cell properties, such as using chlorophyll as a biomass proxy, or over-reliance on genomics or proteomics without integrating physiological processes can lead to misinterpretation of cellular processes. Temporal selective bias emerges from sampling windows that ignore phased physiological demands, including marked daytime changes in photosynthesis and molecular signatures even under constant light. Physical bias arises from poorly controlled experimental conditions such as unstandardized hydrodynamics, altering diffusion boundary layers, gas/nutrient delivery and self-shading. Chemical bias stems from uncontrolled carbonate chemistry (pH, pCO_2_ and dissolved inorganic carbon) and unstable nutrient concentrations. Notably, while changes in carbonate chemistry may leave photosynthetic rates seemingly unchanged due to molecular acclimation (e.g., CO_2_-concentrating mechanisms), this often masks significant shifts in enzymatic activities, such as that of carbonic anhydrase. Crucially, these biases form reinforcing feedback loops: physical disturbance alters chemical availability, altering temporal physiological states, which then modifies molecular responses. By analyzing this multidimensional interplay, this paper provides essential guidance for designing robust experiments, helping researchers avoid the pitfall of “perfectly measured, wholly wrong” conclusions.

## 1. Introduction

The history of science is replete with examples where selective attention—choosing what to measure, when to measure and how to interpret it—has led researchers astray. Here, we take a definition of selective bias as that introduced by selectively choosing or failing to standardize parameters, sampling times and physical factors such as mixing and aeration. In ecological and physiological research, selective bias is manifest when investigators unconsciously favor certain observations while neglecting others that might fundamentally alter causal interpretations [1]. Nowhere is this risk more acute than in studies using experimental cultures of algae, since their growth, along with assimilation of biogenic elements, changes the chemical and physical conditions in the cultures [2]. The interplay of physical forces, temporal dynamics and chemical shifts creates a complex causal web that defies simple univariate analysis.

As a fundamental form of bias, the choice of parameters to measure physiological performance can influence our interpretation of observations. Here, we give two examples. Chlorophyll (especially chl *a*) is frequently used as a proxy for phytoplankton biomass in biological oceanography. This has introduced a bias towards using the abundance of chl *a* as a measure of phytoplankton primary productivity. However, such emphasis on chlorophyll (chl) ignores the fact that chl per cell will change with time in batch growth, decreasing in cases where a nutrient such as nitrogen becomes depleted or increasing due to self-shading effects as cultures become denser. Expressing changes in rates of growth or physiological processes on a chl basis under conditions where chl cell^−1^ changes introduces a bias, leading researchers to overlook alterations in cellular rates or to see changes in cellular properties where none (other than chlorophyll content) exist.

A second example of selective bias in the choice of parameters to examine comes from the use of omics to elucidate biochemical and physiological processes in algae. While the use of omics (primarily genomics, transcriptomics and proteomics) has undoubtedly proved useful in elucidating aspects of algal biology, it is apparent that uncritical use of genomics data can lead to misleading inferences. A good example of this comes from studies on the pathways of photosynthetic carbon assimilation in algae and the debate surrounding the potential use of C_4_ metabolism as a form of CO_2_ concentrating mechanisms (CCMs). For instance, Armbrust et al. [3] reported the presence of genes for the enzymes PEPC and PEPCK (which are involved in C_4_ metabolism in higher plants and a few algae) in the genome of the marine diatom *Thalassiosira pseudonana*. Similar genes have been identified in another diatom, *Phaeodactylum tricornutum* [4] and in the green alga *Ostreococcus tauri* [5]. This has been taken as evidence for C_4_ metabolism in these algae, despite strong biochemical evidence that they display C_3_ and not C_4_ metabolism [6,7]. It seems likely that, in fact, these enzymes, purported to be playing a role in C_4_ photosynthesis, are instead simply involved in anaplerotic β-carboxylations as shown for the coccolithophore *Emiliania huxleyi* [8]. Thus, uncritical reliance on a single technique can lead to major misconceptions about, in this case, the metabolic pathway involved in CO_2_ assimilation.

**Figure 1:**
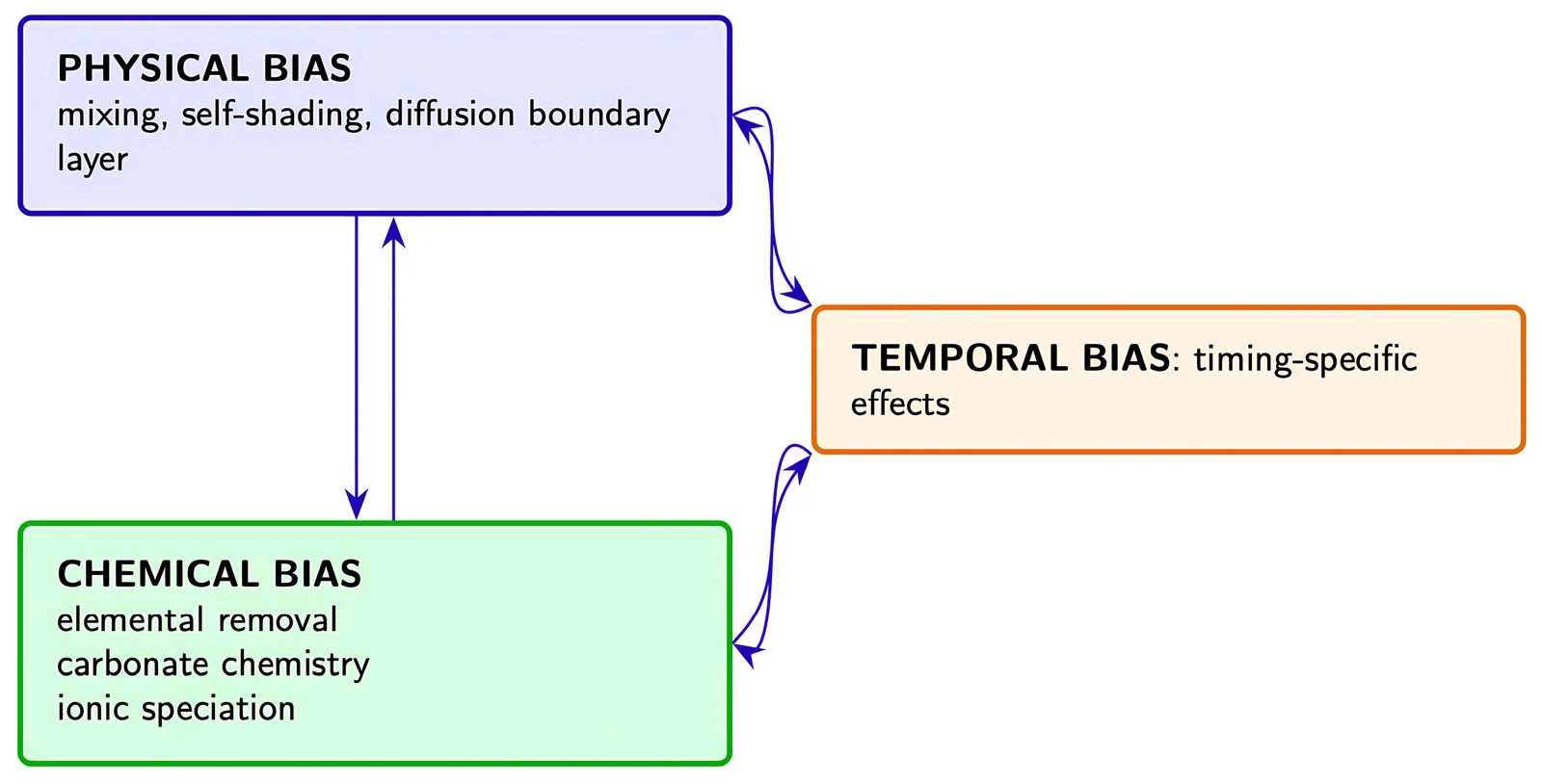
Interplay among physical, chemical and temporal biases. This interconnectedness means that bias analyzed in isolation is bias underestimated. Correcting only for physical bias by standardizing shaking protocols would not eliminate the problem if temporal sampling remains fixed at Day 7. Correcting only for temporal bias by frequent sampling would not eliminate the problem if chemical measurements remain restricted to nutrients with stability of carbonate chemistry ignored. The biases form a system, and interventions must address the system as a whole.

We now consider a deceptively simple experiment designed to test whether a novel nutrient or a material (“Additive A”) promotes the growth of an alga or cyanobacterium. The investigator establishes two kinds of culture: an experimental group receiving Additive A and a control group receiving none. After several days, which is supposed to be long enough for acclimation, the cultured alga exhibits significantly higher cell densities in both groups, being higher at the late phase of the culture supplied with the Additive A. The conclusion appears straightforward: Additive A enhances algal growth. It can be argued that such a conclusion may be fundamentally wrong—not because any measurement was inaccurate but because selective bias operates simultaneously across three dimensions. Physical bias crept in through differential handling (such as using static cultures, or shaking, bubbling, or applying aeration with unknown/different flow rates) and self-shading due to increased biomass. Temporal bias potentially obscured critical phase-specific effects (such as an extended exponential growth phase in order to achieve acclimation). Chemical bias could have masked the true mechanistic drivers (e.g., reduced availability of CO_2_, nutrients and accumulated O_2_ in closed cultures). Physical bias might have disguised the fact that light exposure to each cell decreased dramatically over time due to self-shading along with accumulation of biomass. Worse still, these biases intertwined, each amplifying the others’ effects (Figure 1). By dissecting such a hypothetical experiment, we can illuminate how multidimensional selective bias generates spurious causal inferences and, more importantly, how future studies might avoid these pitfalls.

## 2. Physical Dimension: The Invisible Hand of Hydrodynamics

In our hypothetical experiment, the investigator—unconsciously motivated by the desire to ensure the “promising” culture receiving Additive A thrived—shook the experimental flask for an extra 5–30 s each day or aerated with slightly different flow rates. This seemingly trivial deviation introduces what we term here physical selective bias: the systematic distortion of results through unstandardized physical manipulation. The consequences extend far beyond simple mixing. When a fluid moves relative to a solid surface (the algal cell), a region known as the diffusion boundary layer forms around the cell. Within this layer, molecular diffusion—not turbulent mixing—governs nutrient transport. The thickness of this boundary layer is inversely related to fluid velocity: faster flow or stronger mixing thins the layer, steepening the concentration gradient and accelerating nutrient flux to the cell surface.

Research on periphyton growth has demonstrated that under nutrient-limited conditions, algal biomass accumulation can be regulated more strongly by the diffusion rate through the viscous sublayer than by the absolute nutrient concentration in the overlying water [9]. An increase in fluid velocity has been shown to enhance the absorption of nutrients and growth of a diatom, and the mode of flow modulates such and effect, with turbulent being more effective than laminar flow [10]. Effects of flow type and velocity interact with cell size, morphology, motility, aggregation, culture depth, flask geometry and sensitivity to shear (some dinoflagellates are especially prone to shear stress) [11] to influence the effect of mixing. Such effects are not limited to planktonic microalgae or periphyton; increased current speed of seawater has been shown to enhance photosynthetic rates of macroalgae [12]. The extra shaking or faster stirring with aeration (with inconsistent flow rates) in our hypothetical algal culture likely thinned the boundary layer around cells in the treated flask, accelerating the delivery of not only the target additive but also CO_2_ and nutrients. Clearly, the experimenters need to ensure mixing and aeration regimes are constant across control and treatments.

Crucially, this physical effect interacts with chemical availability in ways rarely considered by experimenters. Trace metals such as iron and manganese—essential cofactors for photosynthetic enzymes—have complex speciation chemistry in aqueous media. In static cultures, these metals may precipitate as oxides or hydroxides, becoming biologically unavailable. Hydrodynamic disturbance can resuspend precipitates, break down concentration gradients, and alter metal speciation through changes in local pH and redox conditions. Thus, the physical act of shaking or aeration does not merely “mix” the culture; it fundamentally alters the chemical environment in ways that confound attribution of growth enhancement to Additive A. Such an Additive A can be taken as the addition of a nutrient, CO_2_ contaminant or some chemical material (such as Metal–Organic Frameworks) to a microalgal culture. For instance, the addition of CO_2_ to cultures of Emiliania huxleyi was shown to either reduce [13] or increase [14] its calcification, leading to completely different implications of ocean acidification (OA). Although interpretation of these differences in response might be attributed to the use of different strains, differences in the means of supplying CO_2_ to the cultures can alter the physiological response of microalgae to targeted CO_2_ levels [15]. Thus, the lack of coherence between the results reported by Riebesell at al. [13] and Iglesias-Rodriguez et al. [14] was possibly because of the modes and/or extents of aeration, such that aqueous pCO_2_ pressure and pH were altered to different extents, leading to deviations from pCO_2_ and pH values projected for the targeted future OA. Differences between the results presented in these two studies can also be attributed to a lack of standardization in culture conditions, timing of acclimation and approaches to data interpretation, all forms of selective bias [16].

## 3. Temporal Dimension: Sampling Window Limitation

The investigator, in our assumed experiment, chose to measure final biomass on Day 7. This decision seems innocuous but introduces temporal selective bias: the distortion introduced by selecting an observation window that fails to capture the full dynamic trajectory of the system. Algal cultures in batch mode progress through distinct physiological phases: lag phase, exponential growth, declining growth, stationary phase and death phase. Each phase is potentially characterized by variable responses to various factors, different metabolic priorities and different responses to experimental manipulations. The physiological state of phytoplankton cells just before nutrient exhaustion differs fundamentally from the state during active exponential growth [17]. Batch cultures of photosynthetic micro-organisms exhibit decreased photosynthetic and respiratory activities along with lower availability of nutrients due to their removal from the external milieu associated with their assimilation [18]. The massive stress responses and cell cycle arrest that occurs when nutrients are fully depleted are less obvious in cells experiencing gradual nutrient limitation due to the algal cells’ physiological acclimation and regulation [19].

In our assumed algal experiment, several temporal scenarios could produce the same Day 7 outcome while implying entirely different causal mechanisms. Scenario 1: Additive A exerts its effect only during the late exponential phase, when bulk nutrients are becoming depleted. Perhaps Additive A contains a micronutrient that becomes limiting only after initial luxury consumption of major nutrients is exhausted. Measuring only final biomass captures this late-phase effect but provides no information about early-phase dynamics. Scenario 2: Additive A slightly inhibits growth during the early exponential phase but contains a nutrient source that becomes available only after enzymatic cleavage by late-stage cells. The final biomass reflects a net effect that obscures the initial inhibition. Scenario 3: Additive A has no direct nutritional role whatsoever. However, experimental cultures achieve higher density by Day 7, leading to increased turbidity, which attenuates light penetration. Cells adapt to lower light by increasing chlorophyll content per cell and adjusting their photosynthetic apparatus. This physiological acclimation—driven by the physical consequence of higher density—then influences subsequent nutrient demand and growth trajectories [2].

Most studies on algal physiology and biochemistry rightly recognize that physiological parameters such as photosynthetic rate differ between exponential and stationary phases and have based sampling times accordingly, usually within the mid to late exponential phase to maximize the biomass available for analysis. However, careful examination reveals that, even within the exponential phase of growth, features such as photosynthetic capacity alter dramatically. This was shown, for instance, many years ago in terms of the dogma surrounding the acclimation of microalgae to low temperatures or low light. At that time, it was believed that algae acclimated to low temperatures by increasing the level of enzymes involved in carbon assimilation to compensate for lower enzyme activity under cooler conditions. Similar hypotheses were current for light acclimation, where it was argued that to compensate for low light availability, algal cells increased their overall capacity for photosynthesis, including their capacity for carbon fixation. Such observations were shown to be a consequence of changes in photosynthetic processes occurring during the course of the exponential phase of growth [20,21].

A further example of temporal-driven bias is afforded by studies on inorganic carbon acquisition mechanisms in microalgae. Changes in cell density lead to changes in the ratio of demand for CO_2_ (driven by photosynthesis) and the supply of CO_2_ (driven by the carbonate chemistry in the culture medium), and this in turn can affect the expression of enzymes such as periplasmic carbonic anhydrase, which can be absent at initially low cell densities and only detectable as culture density increases over time [22]. Similar changes over time have been recorded, for instance, in cultures and natural blooms of the dinoflagellate *Peridinium gatunense* [23,24].

Temporal bias is not only found at the scale of days or weeks but is also apparent when sampling across daily changes in cultures exposed to light/dark cycles. Changes in photosynthetic physiology associated with light/dark cycles (and associated diel changes in oxygen, temperature and pH) are well known [25,26], so timing of measurements in relation to light–dark transitions is critical.

The temporal bias here is not merely a matter of insufficient sampling frequency. It represents a failure to recognize that the meaning of any measurement depends critically on when it is made. Without understanding the phase-specific responses, the investigator cannot distinguish whether, in our theoretical experiment, Additive A acts as a direct nutrient, an indirect modulator of physiology or an artifact of density-dependent feedback.

## 4. Chemical Dimension: The Illusion of Measured Nutrients

Our hypothetical investigator, diligent in quantification, measures nitrate and phosphate concentrations in the culture medium at the beginning and end of the experiment. The data show that experimental cultures consumed more nitrogen and phosphorus than controls. This appears to confirm that Additive A stimulated nutrient uptake. This conclusion exemplifies chemical selective bias: the error introduced by measuring only certain chemical variables while ignoring others that may be mechanistically critical. First, bulk nutrient concentrations in the medium do not necessarily reflect the nutrient experience of individual cells. As diffusion boundary layer theory makes clear, the concentration at the cell surface—the concentration the cell actually “sees”—can differ substantially from the well-mixed bulk concentration [12]. In static or poorly mixed cultures, cells may experience effective nutrient limitation even when bulk concentrations appear sufficient. Second, nutrient consumption does not equal nutrient utilization for growth. Cells may take up nutrients in excess of immediate metabolic requirements, storing them internally as polyphosphates, cyanophycin granules or lipid bodies. This luxury consumption decouples nutrient depletion from biomass production. An additive that stimulates storage without stimulating growth would appear to enhance “uptake” in bulk measurements while producing misleading conclusions about growth promotion. Third, and most critically, selective measurement of nitrogen and phosphorus ignores the potential role of trace elements—iron, manganese, zinc, cobalt, molybdenum and vitamins—that often become limiting in later growth stages. Multiple stressors—flow regime, temperature and light—interact to control phytoplankton dynamics in ways not captured by simple nutrient measurements.

In our above assumed experiment, the extra shaking or inconsistent aeration in experimental cultures might have resulted in different levels of CO_2_ supply from the air, altering CO_2_ availability or the ratio of CO_2_ to O_2_ in water, that would affect photorespiration and other physiological performance [27].

## 5. The Interplay of Dimensions: A Causal Web

The most insidious aspect of selective bias in our hypothetical experiment is not the operation of each dimension individually, but their mutual reinforcement. Physical, temporal and chemical biases do not simply add their effects; they interact multiplicatively to generate a coherent but entirely spurious causal narrative (Figure 1). Consider the causal pathways that remain invisible to the selectively observant investigator:

Physical → Chemical: Enhanced shaking or stronger aeration (physical) increases CO_2_ supply and alters pH and thereby trace metal speciation (chemical). The resulting change in the chemical environment affects cellular physiology.

Chemical → Temporal: Altered trace metal or macronutrient availability (chemical) may have negligible effects during the early exponential phase when luxury reserves suffice but become growth-limiting during the late phase when internal stores are depleted. This creates a temporally dependent chemical effect that only manifests at specific sampling windows.

Temporal → Physical: Higher cell density achieved by the late phase (temporal effect) increases culture turbidity, altering the light field experienced by cells. Reduced light (physical effect) induces photoacclimation responses that modify cellular stoichiometry and nutrient demand, feeding back to chemical dynamics.

Physical 7. Sampling point, experimental and control cultures are in fundamentally different physiological states, making direct comparison meaningless.

## 6. Toward Multidimensional Experimental Design

**Figure 2:**
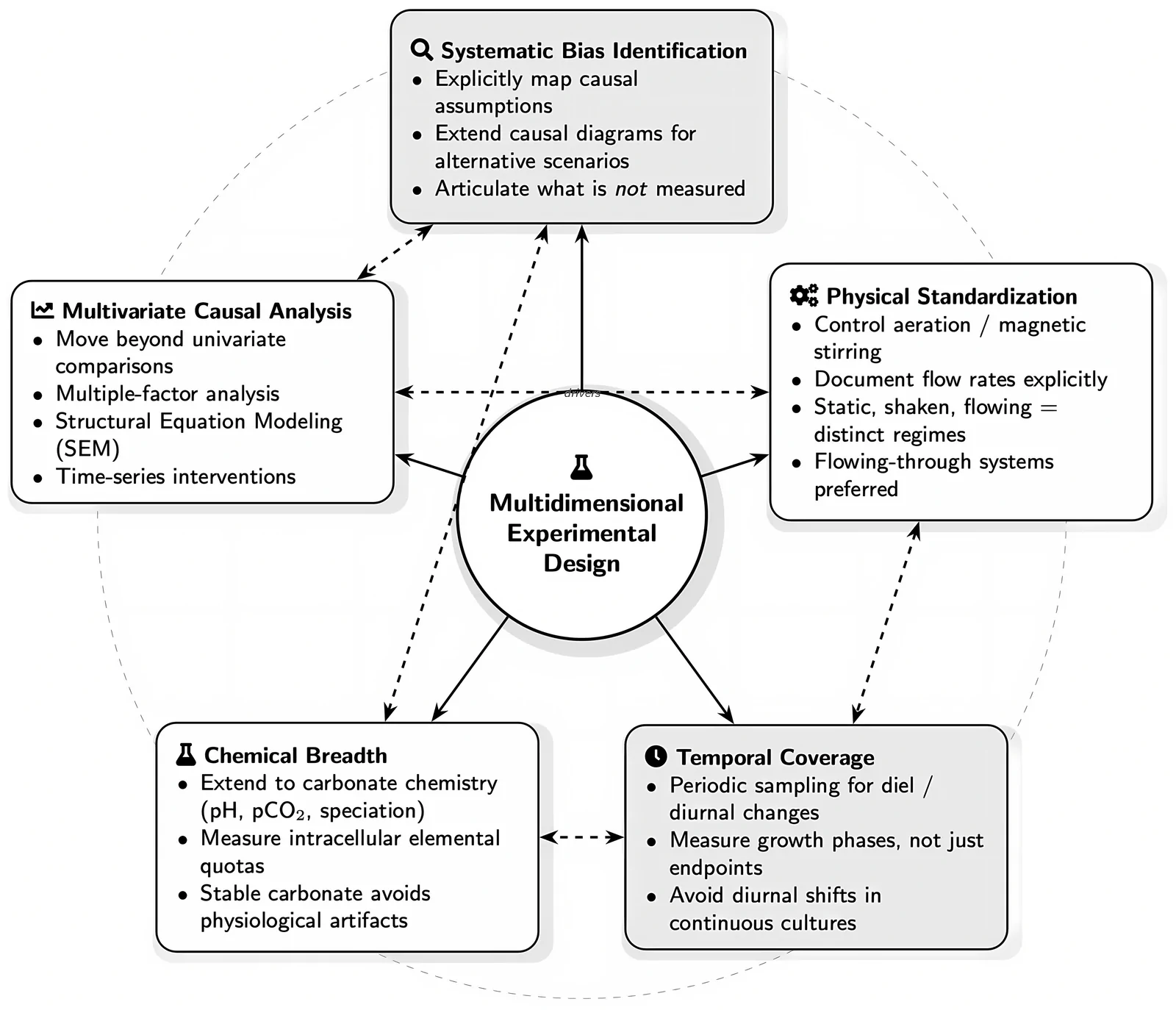
Multidimensional experimental design with systematic bias identification to avoid potential biases. Note, continuous or semi-continuous cultures (maintaining exponential growth) are recommended for maintaining a stable chemical environment.

Recognizing the multidimensional nature of selective bias suggests concrete strategies for experimental design for working with cultures of algae or cyanobacteria (Figure 2):

1. Physical standardization with documentation. Fluid dynamic conditions should be explicitly controlled and reported. If mixing is not the variable of interest, standardized aeration or magnetic stirring should be used with documented rates. Growing the photosynthetic organisms in flow-through systems would be more desirable [12]. “Static”, “shaken” and “flowing” should be recognized as representing different hydrodynamic regimes, not neutral alternatives. For some microalgae, such as dinoflagellates that are vulnerable to stirred forcings, dialysis tubes can be employed to enclose them in mixed or aerated water to achieve chemical stability [28].
2. Temporal coverage, not just endpoints. Periodical samplings should be implemented to capture diel or diurnal differences in physiology or molecular performances. The time-spans would differ for different experimental approaches or purposes. As research on high-frequency water quality monitoring has demonstrated, weekly sampling may be wholly inadequate to identify causal drivers of phytoplankton dynamics; hourly data reveals thresholds and interactions invisible at finer resolution. Not only final biomass but also growth rates during the exponential phase, timing of stationary phase onset and duration of each phase need to be measured if batch cultures are run. With continuous or semi-continuous cultures, measurements should be designed to avoid diurnal or diel changes or standardize sampling times so that comparisons could be fairly made. The potential effects of repeated sampling of cultures on features such culture volume, surface area:volume relationships and gas exchange should also be noted.
3. Chemical breadth and intracellular assessment. Chemical measurements should be extended beyond bulk nutrients to include carbonate chemistry parameters as well as intracellular elemental quotas. Stability of carbonate chemistry in the cultures ensures stable, pCO_2_, pH and ionic speciation. Ignoring these could bring about significant artifacts in molecular and physiological analysis.
4. Systematic bias identification. Frameworks should be adopted for systematic identification of multiple potential biases, mapping causal assumptions explicitly and extending causal diagrams to represent alternative scenarios under which biases might arise. This approach forces investigators to articulate what they are not measuring and why those omissions might matter.
5. Multivariate causal analysis. It should be a given that any experiment needs adequate biological replication. Taking multiple samples from the same flask/culture unit constitutes a pseudo-replication; using an adequate number of independent biological replicates is thus essential. Variables such as inoculum density and culture history should be standardized; treatments should be randomly allocated across culture units and culture positions should be rotated where feasible. It is recommended that statistical analysis moves beyond univariate comparisons to analytical methods that can accommodate interacting drivers. Multiple-factor analysis, structural equation modeling and time-series interventions can help disentangle the web of physical, temporal and chemical influences.

## 7. Conclusions

It is commonly known that, for a cyanobacterium or microalga influenced by environmental changes, the cells can detect the change (seconds to minutes), transcribe the new mRNA (faster in cyanobacteria than in microalgae) and translate that mRNA into protein. Therefore, for a responsive physiological trait to be measurable, it takes longer than the underlying molecular responses, and it takes longer to occur in eukaryotic algae than in cyanobacteria [29,30].

The hypothetical algal cultivation experiment described here contains no fraudulent data, no faulty equipment and no deliberate misrepresentation. Every measurement is assumed to be accurate. Every observation is real. Yet, the conclusion—that Additive A promotes algal growth—may be entirely wrong. This paradox arises from selective bias operating across three interconnected dimensions. Physical bias enters through differential handling that alters diffusion boundary layers and nutrient delivery. Temporal bias obscures phase-specific effects by fixing observation at a single endpoint. Chemical bias masks mechanistic drivers by measuring only a subset of relevant variables. And, these biases do not remain separate; they intertwine, each amplifying the misleading signals generated by the others.

In order for algal researchers to avoid selective bias as much as possible, recommendations are provided in Table 1. They should be aware of the fact that the lesson extends far beyond phycology. In any complex biological system where physical forces, temporal dynamics and chemical transformations interact, selective observation creates the illusion of simple causation. The remedy is not merely more measurements, but measurement strategies guided by explicit causal models that acknowledge what might be missed. Recognizing the multidimensional nature of selective bias is the first step toward experiments that discover real causal relationships rather than confirming our selective expectations. For algal research, continuous or semi-continuous cultures are recommended for studies on physiological and/or molecular responses to a driver (such as temperature), so that the algal cells can be sustained in a stable exponential growth phase [2]. Although semi-continuous and continuous cultures have their own set of strengths and weaknesses and may not be appropriate for exploring some experimental questions, they do offer a way to minimize selective bias due to chemical and physical changes along with accumulation of biomass.

## Figures and Tables

**Table 1:** Recommended guides for experimental designs in algal and cyanobacterial research.

Groups	Main Selective-Bias Risks	Physical Standardization	Chemical Standardization	Temporal Design
Macroalgae	Boundary-layer thickness; self-shading; different parts used	Same levels of mixing, flowing currents and/or light	Monitor carbonate system, nutrients	Acclimate before sampling at same time of day
Microalgae/cyanobacteria	nutrient depletion; self-shading; unequal shaking/aeration	Documented standardized shaking, aeration or magnetic stirring; position	Continuous, semi-continuous cultures or dilute batch cultures; biogenic elements	Periodic sampling at same diel time; consider mRNA/protein response times
Phytoplankton assemblages	Community composition shifts; grazing; patchiness; enclosure artifacts; unequal mixing/light/nutrients	Same mixing regime, depth, light field, enclosure material	Monitor nutrients, DIC, pH, pCO_2_, O_2_; avoid contaminations	Biomass, growth and photosynthesis; not just final chlorophyll
Mixing-sensitive dinoflagellates	Shear/stirring damage; altered behavior/vertical migration; static vs. mixed differences	Avoid vigorous stirring; use dialysis bags suspended in mixed/aerated water; slow rotation	Dialysis exchange with external medium; monitor inside/outside pH, pCO_2_, DIC, nutrients	Sample at same diel time; account for vertical migration and diel cycles; periodic sampling

## Data Availability

Data sharing is not applicable to this article as no new datasets were generated or analyzed during the current study.
